# Multi-InDel Analysis for Ancestry Inference of Sub-Populations in China

**DOI:** 10.1038/srep39797

**Published:** 2016-12-22

**Authors:** Kuan Sun, Yi Ye, Tao Luo, Yiping Hou

**Affiliations:** 1 Institute of Forensic Medicine, West China School of Basic Science and Forensic Medicine, Sichuan University, Chengdu, P.R. China; 2 Laboratory of Infection and Immunity, School of Basic Medical Sciences, West China Center of Medical Science, Sichuan University, Chengdu P.R. China

## Abstract

Ancestry inference is of great interest in diverse areas of scientific researches, including the forensic biology, medical genetics and anthropology. Various methods have been published for distinguishing populations. However, few reports refer to sub-populations (like ethnic groups) within Asian populations for the limitation of markers. Several InDel loci located very tightly in physical positions were treated as one marker by us, which is multi-InDel. The multi-InDel shows potential as Ancestry Inference Marker (AIM). In this study, we performed a genome-wide scan for multi-InDels as AIM. After examining the F_ST_ distributions in the 1000 Genomes Database, 12 candidates were selected and validated for eastern Asian populations. A multiplexed assay was developed as a panel to genotype 12 multi-InDel markers simultaneously. Ancestry component analysis with STRUCTURE and principal component analysis (PCA) were employed to estimate its capability for ancestry inference. Furthermore, ancestry assignments of trial individuals were conducted. It proved to be very effective when 210 samples from Han and Tibetan individuals in China were tested. The panel consisting of multi-InDel markers exhibited considerable potency in ancestry inference, and was suggested to be applied in forensic practices and genetic population studies.

A fair amount of Ancestry Information Markers (AIMs) have been proposed for various purposes including detecting stratification in biomedical studies^1^,^2^ and determining an individual’s ancestry from a forensic context^3^,^4^,^5^,^6^,^7^,^8^,^9^,^10^,^11^,^12^,^13^,^14^. In the genotype-phenotype studies, such as the popular genome-wide association studies (GWAS), the presence of uncontrolled population structure may lead to false-positive or false-negative results^15^,^16^,^17^,^18^,^19^. To eliminate the adverse impact to the outcomes, AIMs are used to detect and control the potential population stratification derived from genetic ancestry. Besides, in the forensic practices, ancestry information could provide vital clues and narrow down the investigation scope, when existing profiling is unsuccessful, no DNA database matches are obtained or in the absence of reliable eyewitness testimony. In reality, samples from forensic contexts are always challenging, with limited quantity or poor quality. Therefore, a small-scale but robust panel of AIMs is preferred. In fact, it is the capability of discerning subtle differences between closely related populations, which may share many similarities in evolution or/and close residences in geography, that matters in the practical police investigations. Such researches^20^,^21^,^22^ have been reported for some special regions. Yet it is absent in eastern Asian. Regarding to this range, Chinese Han population is naturally the ideal study object. As the largest ethnic group in the world, it comprises about 20% of the global human population. Chinese Tibetan population also deserved to be studied for the unique genetic characteristics inherited from their ancestries, which adapt the Tibetan people to the plateau environment. Researches on ancestry analyses of these two ethnic populations are undoubtedly beneficial to clinical association studies as well as forensic identifications. No results have been published yet.

Small molecular regions comprised of two or more SNPs have been reported to convey more identity and ancestry-related information^9^,^23^. Systems consisting of such multi-allelic haplotype markers have also been defined and advocated developing for forensic use, because they could serve to identify relatives with higher probabilities than simple di-allelic SNPs^24^. Replacing SNPs with InDels, a novel concept termed multi-InDel has been reported to behave well in parentage tests and to be hopefully used for forensic applications in the near future^25^,^26^. Meanwhile, the prospect of this new kind of markers in the population genetic studies deserves to be explored and discovered, especially that regarding population stratification and ancestry inference. Reasons are listed as below: (1) The looselinkage^9^ multi-InDel markers adopted shows conservation of evolutionary lineages. They have evolved from the ancestral human haplotype without evidence of recurring recombination; (2) Different from the traditional complete linkage disequilibrium (LD), historic recombination may occurred, while the recombinants have drifted to sufficiently high frequency to become common haplotypes; (3) With high inter-population variability, InDel is perfectly suitable for population genetic researches. Furthermore, the feasibility and practicability of this research are significantly enhanced thanks to the advantages of InDels including, ubiquity throughout the genome, second only to SNPs; well characterized^27^,^28^; low mutation rates; simplicity of laboratory analysis, like routine capillary electrophoresis (CE) typing; short amplicon strategy that could be adopted for degraded DNA samples; as well as the possibility of genotyping several markers in a single PCR multiplex reaction.

Based on above, we made a bold speculation that multi-InDel might do well in the study of population stratification, even ancestry inference in admixed populations. And we proved it through an example of a multiplex assay including 12 multi-InDel markers in this paper.

## Materials and Methods

### Ethics Statement

Human blood samples were collected upon approval of Ethics Committee at Sichuan University, P. R. China. A written informed consent was obtained from each participant in this study. This study was approved by the Ethics Committee of Sichuan University, P. R. China.

### Sample Collection and DNA Extraction

A total of 210 blood samples were collected, among which 110 samples from Chinese Tibetan individuals were collected in Tibet Autonomous Region and 100 samples from Han individuals were collected in our laboratory. All the samples were obtained with written inform content and self-declared ancestry information according to quality control procedures. Genomic DNAs were extracted from peripheral blood samples or bloodstain samples using the Chelex-100 method as described by Walsh *et al*.^29^. DNA was quantitated using the NanoDrop 1000 Spectrophotometer (Thermo Fisher, MA, USA). In addition, samples for known cryptically related individuals were removed^30^.

### Marker Selection

Candidate Indels were chosen from the 1000 Genomes database^31^,^32^ (http://www.ncbi.nlm.nih.gov/variation/tools/) when the following principles were both met: (a) F_ST_ > 0.4 when comparison was performed between any two of the five continental populations (AFR, African; EAS, East Asian; EUR, European; SAS, South Asian; AMR, Admixed American); and (b) F_ST_ > 0.15 when comparison was performed between any two of the five sub-populations in EAS (CHS, Southern Han Chinese, China; CHB, Han Chinese in Beijing, China; JPT, Japanese in Tokyo, Japan; KHV, Kinh in Ho Chi Minh City, Vietnam; CDX, Chinese Dai in Xishuangbanna, China). Based on these core loci, additional criterions were applied to construct multi-InDel markers, including: (i) the physical distance between the InDels in one multi-InDel marker was less than 250 bp for the formation; (ii) there were at least two haplotypes for each multi-InDel marker to ensure the heterozygosity; and (iii) the amplicon length was less than 300 bp.

The scanning procedure focuses mainly on the formation of combined InDel markers and the high level of F_ST_ parameters for studied populations. Specific steps include (1) calculating F_ST_ values for subject populations as described by Weir and Cockerham^33^; (2) sorting F_ST_ and screening loci with standard parameters meeting the preset criteria; and (3) checking flanking regions for proper combinations of InDels with extremely short physical distances.

Flanking regions of the selected multi-InDel markers were also obtained from the University of California Santa Cruz Genome Browser (Human February 2009 Assembly; GRCh37/hg19) at http://genome.ucsc.edu/. Sequences were checked for variants and polymorphism structures, which are likely to interfere with primer design or data analysis.

### Primer Design and Optimization

Primer designing was performed with the Primer Premier v5.0 (PREMIER Biosoft, Palo Alto, CA, USA), applying the following criteria: PCR product size from 70 to 300 bp; Tm values from 52 to 60 °C and GC content from 30 to 60%. The “PIGtail” or partial tails of the original GTTTCTT nucleotide sequence were added to the 5’end of the unlabeled primers to promote full adenylation^34^. The obtained primer pairs were examined for potential interactions with each other using AutoDimer software^35^. They were also checked for non-specific hybridizations in other genome regions using the National Center for Biotechnology Information (NCBI) Basic Local Alignment Search Tool (BLAST) at http://blast.ncbi.nlm.nih.gov/Blast.cgi. All the markers were then schematically organized by expected amplicon length and assigned into four different dye-labeling fluorochromes (FAM, HEX, TAMRA, and ROX) (Applied Biosystems, Foster City, CA).

Optimizations of the multiplex assay were performed on the basis of primer concentrations and peak heights. After that, 210 collected Chinese samples from Han and Tibetan individuals were genotyped with the developed PCR system. Based on the size information of each haplotype, Panel and Bin files were programmed.

### PCR Setup and CE Genotyping

The PCR system was a 12.5 μL reaction volume containing 1× Qiagen multiplex PCR master mix, 1 × Q-Solution, 2 μL primer mix and 0.5–2 ng of template gDNA. Thermal cycling conditions consisted of an initial step at 95 °C for 15 min; 32 cycles at 94 °C for 30 s, 56 °C for 90 s, and 72 °C for 60 s; and a final extension at 60 °C for 45 min. For the Tm value, 54 °C, 56 °C and 58 °C were chosen for testing.

PCR products were analyzed by mixing 1 μL of each amplified product with 9 μL in a 17:1 mixture of Hi-Di formamide (Applied Biosystem, Foster City, CA) and SIZ 500 (AGCU Co, China) for CE. Fragment detection and separation were performed on ABI Prism 3130 Genetic Analyzer (Applied Biosystems, Foster City, CA). Genotyping data was analyzed with GeneMapper v3.2.1 software (Applied Biosystems, Foster City, CA). Default setting of peak height (above 200 RFU) was applied for the analysis. The positive controls (9947A) and negative controls (DI water) were performed by the same reaction condition.

### Analytical Method

Haplotype frequencies were determined by counting. Analyses of genetic parameters such as F_ST_ values were performed with vcftools_0.1.12b^36^. PCA analysis based on the genotypes of the 12 multi-InDel markers was operated with SPSS 16.0.

Individual ancestry components were primarily examined via model-based clustering algorithms implemented in STRUCTURE 2.3.4^37^, which was based on a Bayesian Markov Chain Monte Carlo algorithm. Analyses were performed with five replicates from K = 1 to K = 7 using the no-admixture model and correlated allele frequencies (100,000 burn-ins iterations and 10,000 MCMC repeats) to estimate the selected 12 multi-Indel markers. Structure Harvester^38^ was applied to estimate the optimum K value. Results of ancestry proportions were compared to the self-identified ancestry. We ran STRUCTURE for increased K values to observe if the multi-InDel panel had the potential to discern more subtle structures in the selected samples.

Inference of ancestry affiliation was estimated using the *Snipper* Classify using frequencies option (at: http://mathgene.usc.es/snipper/ “Classification with the 32 STR training set or a custom Excel file of frequencies”), a direct link provided by the *Snipper* portal. Ten out of the 210 collected Chinese samples from Han and Tibetan individuals were randomly chosen as blind trials. The rest were developed to the training set, an Excel-based data input system using one worksheet per marker (alleles as columns, populations as rows) listing allele frequencies in each cell. Ancestry assignments were performed to test the accuracy of this inference system with the 12 multi-InDel markers.

### Quality Control

The main experiments were conducted at the Forensic Genetics Laboratory of West China School of Basic Science and Forensic Medicine, Sichuan University, P.R. China, which is an accredited laboratory by ISO 17025. All the methods were carried out in accordance with the approved guidelines of Institute of Forensic Medicine, West China School of Basic Science and Forensic Medicine, Sichuan University, P.R. China.

## Results and Discussion

### Multi-InDel Markers Chosen for distinguishing Ethnic Populations

**Figure 1:**
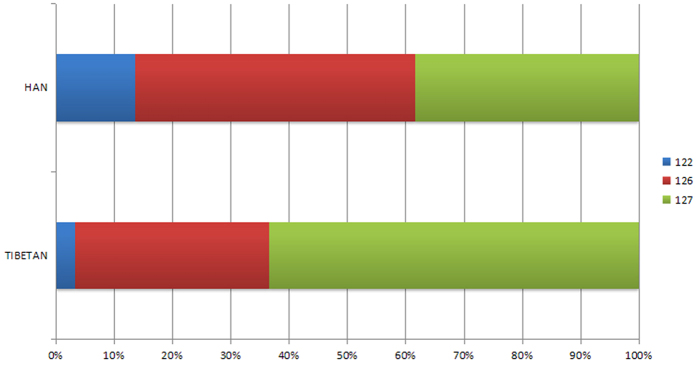
Haplotype frequency distributions of Multi-Indel No.5 in Han and Tibetan populations in China. Blue bars denote allele122, red and green bars alleles 126 and 127, respectively.

After data analysis and loci screening based on data resource from 1000 Genomes Database, experiment validations were additionally conducted to eliminate some loci with which we cannot obtain stable results. Among various reasons, successful multiplex PCR for all selected markers is the first to be taken into consideration. Primers for each locus were initially tested in a singleplex PCR reaction to evaluate the performance. The criteria for primer “failure” are defined as those that produce profiles that exhibit incomplete adenylation, the presence of PCR artifacts, low signal, nonspecific products, or no PCR products at all. Once the successful primers at each locus were determined, those were equally combined together for a primer mix of 0.1 μM at first. Based on the results of genotyping profiles, the optimization of each primer’s concentrations in the final primer mix was performed. Furthermore, successful PCR without stable performance in the replicated experiments are eliminated, too. Situations are filtered out such like (1) repeated sequences appear in the flanking region of candidate loci; (2) core sequences are variable; and (3) SNPs are detected in the primer binding regions. A total of 12 multi-InDel markers picked into the panel are all robust, accurate, specific and sensitive to ensure the integral efficacy of the panel as a whole. Detail information is presented in Table 1, including the rs numbers, localizations, and alleles of the contained InDels. Multi-InDel No.5 is an example of two InDels (rs568000255 and rs148177611, 65 bp apart) defining three haplotypes in the 210 collected individuals, namely 122, 126, and 127 according to their amplicon size, as illustrated in Fig 1. For each haplotype, distributions in the two sub-populations are strikingly different, which guarantees the capability of the marker to differ one population from the other. Coupled with the other 11 multi-InDel markers, especially that containing more InDels, the assay developed will be sufficiently informative for ancestry inference.

Unlike other AIMs^2^,^12^,^20^,^21^,^39^, we chose InDel as the core unit of the markers. InDel is suitable for population analysis, since the allele distributions show significant differences in different geographic regions^40^,^41^. Studies based on simple InDels have proved its great potential in ancestry inference researches^3^,^7^,^42^,^43^. F_ST_ and I*n* are commonly used to measure the ancestral information of AIMs. However, a high correlation has been found between these two parameters^2^. In this study, we treated F_ST_ value as the leading factor among the selecting criteria, since it concerns the power of differentiation more directly and typically. Population-specific markers were preferred. They included loci with a polymorphism detected in one population but absent in the other or those with a common allele in one population that was rare in others. In the process of selection, we arbitrarily took F_ST_ = 0.15 as the lower limit when sub-populations within EAS were compared to each other so as to balance the number of markers and the power of each marker. In this way, the total power of differentiation was guaranteed. Additionally, continental populations were compared with each other for the warranty of real difference in the allele distributions, which is essential to the aim of ancestry inference.

For multi-InDels, multiple haplotypes with heterozygosity are defined depending on the accumulation of the variants at the different sites, the occurrence of rare crossovers historically, the vagaries of random genetic drift, and/or selection^23^. Profiles with multiple haplotypes provide more information than that with simple di-allelic markers in ancestry inference as well as individual identification or even identifying biological relatives. Generally, increasing the number of genetic markers applied is a conventional approach to achieve more reliable results. While thanks to the sufficient heterozygosity, similar effectiveness could be obtained with a small-scale of multi-InDel markers without any sacrifice of stability or ancestry information.

### Twelve Multi-InDel Markers in One Panel

The final panel was composed of 12 multi-InDel markers, which can be amplified with reproducibility in a fast and easy PCR multiplex reaction followed by electrophoresis. The primer information such as sequences, Tm values and concentrations in the final multiplex was listed in Table 2. The panel proved to be capable of amplifying all of the markers in samples containing different amounts of largely variable DNA (0.1–10 ng). In this effective detection range, full and clear genotyping profiles were obtained from all tested samples. Signal strength variation and stutters appearing in some individual samples with extremely high/low DNA concentration, albeit slightly higher than those of kit markers, did not interfere markedly with profile interpretation. Moreover, the length of a multi-InDel marker was restricted to smaller than 300 bp. Supposing a genome-wide average of ~1% recombination per mega base and no recombination hot spots within the locus, an extent of <300 bp for a multi-InDel marker is projected to bring the recombination rate down to a value comparable to the mutation rate for SNPs. Very rare historical recombination events remained to assume identity by descent within a family after the exclusion of recombination hot spot. At the same time, the small-amplicon strategy diminishes allele and locus dropout and improves the chances of successful analysis of challenging samples with degraded DNA.

### Validation of the 12 Multi-InDel Markers as AIMs

Several strategies including ancestry component analysis with STRUCTURE, ancestry affiliation prediction with *snipper* as well as unsupervised cluster analysis with PCA were performed to estimate the developed panel.

**Figure 2:**
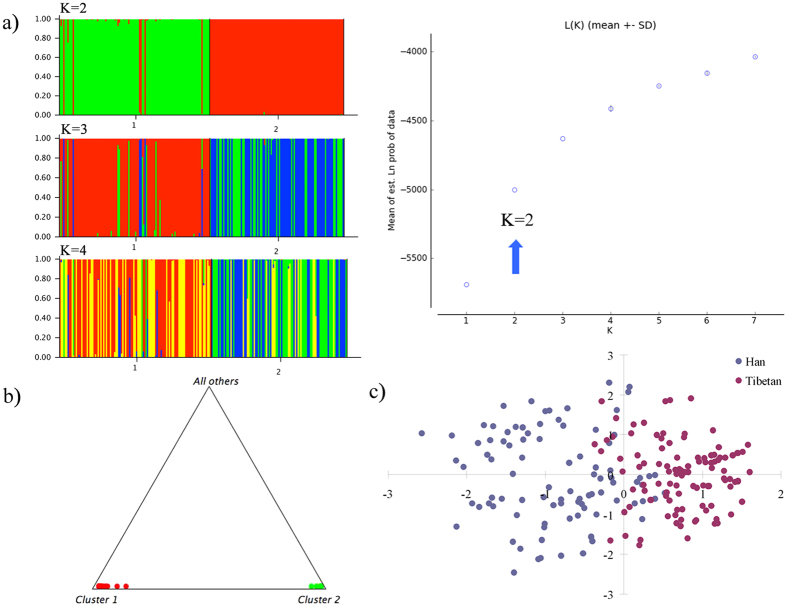
Ancestry component analysis result for 210 Chinese samples from Han and Tibetan individuals with the 12 selected multi-InDel markers. (**a**) Bar plot of STRUCTURE runs for K = 2–4. Chinese samples from Tibetan and Han individuals are marked by Arabic numerals 1 and 2, respectively. The bars with different colors represent different ancestry origins from the analyses. Structure Harvester estimated the optimum K value to be K = 2 (marked by blue arrows in the Ln estimated probabilities plots). (**b**) Triangle plot of STRUCTURE runs for K = 2. (**c**). 2-dimensional plot of PCA analysis. The first two principal components (PC1 vs. PC2) demonstrate the population stratification in the tested samples. PC1 is represented by the X axis, and PC2 is represented by the Y axis.

210 Chinese samples from Han and Tibetan individuals were combined to perform an ancestry component analysis. STRUCTURE runs for K = 2–4 are shown in Fig 2. Optimum K value was estimated to be K = 2 by Structure Harvester. Bar plot of K = 2 analysis reveals that all the collected samples can be primarily separated into two clusters, with one color bar representing each continental origin. The two principal components dominate in Chinese Tibetan and Han, respectively. Additionally, a slight flow representing Han component appears in the supposed Tibetan region, which may be explained by the historical features of Han, such as complex ancestral origin, long history of interaction with surrounding ethnic groups and recent migrations. Nevertheless, Chinese Tibetan displays predominantly component by itself, which is demonstrated more clearly by the triangle plot. No more subtle stratification was observed by further increasing the K value. Compared with previous researches^13^, we distinguish sub-populations in a further step.

To interpret the inference results in a comprehensive and straightforward manner, we performed an assignment test using *snipper*. All the trial samples were accurately assigned into their self-identified populations based on the training set. No misclassification occurred during the test of ancestry inference. *Snipper*, a Bayesian ancestry analysis system, was designed for forensic AIM-SNPs initially. Ancestry assignments of SNP genotype profiles are derived from the ratio of the two highest likelihoods from multiple population comparisons with likelihoods calculated from allele frequencies estimated using training. Later, *Snipper* has been improved to handle the multiple alleles of STRs by accepting training sets of user-input allele frequencies rather than genotypes^6^. Results from this web-based tool are clear and easy to understand with the familiar way we adopted in the routine forensic practice.

Result of the PCA test is shown in Fig 2 too. 210 Chinese samples from Han and Tibetan individuals are spread along PC1 (the X axis), suggesting population stratification in tested samples, although not so pronounced as that in the ancestry component analysis through STRUCTURE. The main tendency clearly indicates two divergent genetic structures though a little overlap appears between them. The clustering of the collected samples was mainly explained by PC1 and PC2, while other PCs were much less informative. And no discernible structure was detected in other combinations of PCs. Compared with that of Tibetan, samples of Han individual are more dispersed in the 2-dimensional PCA plot, reflecting the complex genetic background of Han. Unlike model-based algorithm STRUCTURE implemented or Bayesian analysis *snipper* adopted, PCA is a classical nonparametric linear dimensionality reduction technique, extracting the fundamental structure of a dataset without the need for any modeling. It has recently been shown to be powerful for the identification of population structure and the correction of stratification in the setting of association studies^44^,^45^. Coupled with a clustering tool, it can also be used for inferring population clusters and assigning individuals to sub-populations^46^.

In sum, using the 12 multi-InDel markers, we obtained a perfect classification in ancestry component analysis with STRUCTURE. Besides, ancestry affiliation prediction with *snipper* also assigned trial samples to their correct ethnicity in our test. Results of PCA clustering showed that the separation of the two ethnic populations was clear and definite.

## Conclusion

A multiplex assay with 12 multi-InDel markers as AIM was developed in this study. In validation, 210 Chinese individuals from Han and Tibetan populations were separated into two clusters in accordance with their self-declared ethnic information both in model-based analysis by STRUCTURE and in the unsupervised way, PCA. The ethnic affiliations tests using the web-based tool- *snipper* revealed no mismatch. By constructing multi-InDel markers as the AIMs, we provided a set of markers with improved performance in distinguishing and clustering two closely resided sub-populations. It is a helpful and beneficial exploration for applications on ancestry inference, not only in forensic practices but also in population genetics.

## Additional Information

**How to cite this article**: Sun, K. *et al*. Multi-InDel Analysis for Ancestry Inference of Sub-Populations in China. *Sci. Rep.* **6**, 39797; doi: 10.1038/srep39797 (2016).

**Publisher's note:** Springer Nature remains neutral with regard to jurisdictional claims in published maps and institutional affiliations.

## Figures and Tables

**Table 1:** General Information of the 12 Multi-InDels Chosen for the Test.

ID	Loci	Chromosome	Position	Allele
5	rs568000255	12	111390389	-/T
	rs148177611		111390454	AGAA/-
7	rs587641570	14	106091681	-/TGGGCACGG
	rs35171885		106091743	A/-
9	rs587619205	14	106132379	AGG/-
	rs72033070		106132423	TG/-
	rs587723906		106132479	AGG/-
13	rs576201582	14	106267547	AA/-
	rs571755931		106267673	-/GAACCACGGACAGC
17	rs367879758	6	29893482	T/-
	rs145760005		29893549	AAAC/-
24	rs9281938	6	32576282	-/A
	rs66715534		32576384	AAG/-
29	rs71848820	6	29906220	TACC/-
	rs535382238		29906247	-/AC
	rs372209280		29906267	AA/-
	rs551483768		29906274	AATT/-
	rs113403777		29906356	AT/-
30	rs113251661	6	29920335	-/T
	rs28993377		29920400	CT/-
	rs139015681		29920487	AAAG/-
31	rs535742949	6	29921529	-/G
	rs111867975		29921542	C/-
	rs139686584		29921679	GAA/-
38	rs573698459	11	101349079	T/-
	rs113869189		101349205	-/TTCCCCTCCTCTTG
	rs572328951		101349248	AAAT/-
46	rs58621233	14	106175037	-/ATGCCATG
	rs59809572		106175040	-/CCAGGAGGACAG
	rs587739978		106175045	-/G
52	rs9279904	6	32608168	AT/-
	rs139765606		32608178	A/-
	rs146682150		32608282	-/A
	rs531139227		32608334	AAA/-
	rs140779686		32608357	-/A
	rs148817405		32608392	-/T
	rs67106675		32608459	A/-

**Table 2:** Primer Information of the 12 Multi-InDels Multiplex.

ID	Loci	Allele	Primer-F	Tm-F	Primer-R	Tm-R	Amplicon Size	Primer Concentration (μM)
5	rs568000255	-/T	TCTGATCAAAGATCCCAGA-HEX	62.9	GTGTTAGTTCTCCAACTTTATTAT	59.7	128	5
	rs148177611	AGAA/-						
7	rs587641570	-/TGGGCACGG	TGGATGCAGGCTACTCTA	61.6	CTCCCTCAGCTCAGACAC-HEX	63.1	176	0.8
	rs35171885	A/-						
9	rs587619205	AGG/-	AGTGTCAGGGACAGGAGG	64.9	CTACACTGTCTTCTCGTCTCC-HEX	63.1	147	0.8
	rs72033070	TG/-						
	rs587723906	AGG/-						
13	rs576201582	AA/-	ATAGAGAGGCGCTGGGTAT	65.4	CACTGTTCCACATTTGTCTT-HEX	62.4	251	4
	rs571755931	-/GAACCACGGACAGC						
17	rs367879758	T/-	TATGTATCAAGGGGCCAAAG	66.5	TGGAGGCGTAGAGACAGG-HEX	66.4	192	8
	rs145760005	AAAC/-						
24	rs9281938	-/A	TGAAAAGAAAATTGCTGTAATG-TAMRA	63.8	TCTTTTCCATCATTGTCC	60.4	160	6
	rs66715534	AAG/-						
29	rs71848820	TACC/	AGGTGCAGCAAACCAAC-FAM	64.5	CACCTCTAGAAAGGAACAGTATC	62.9	228	1.5
	rs535382238	-/AC						
	rs372209280	AA/-						
	rs551483768	AATT/-						
	rs113403777	AT/-						
30	rs113251661	-/T	CGTGTTCCTAGATTGGAGTTAA	65	CGTATAATAATGCCTTTACAATCA-FAM	64.2	247	3
	rs28993377	CT/-						
	rs139015681	AAAG/-						
	rs535742949	-/G						
31	rs111867975	C/-	GGTGACAGGGTGAGACTCT	63.9	ATATCCCACGTGGCTGT-ROX	63.5	250	4
	rs139686584	GAA/-						
	rs573698459	T/-						
38	rs113869189	-/TTCCCCTCCTCTTG	GGGATCAAATTTGTAACAG	59.3	ATCATTTGTGCCAAGAATT-TAMRA	61.9	244	8
	rs572328951	AAAT/-						
	rs58621233	-/ATGCCATG						
46	rs59809572	-/CCAGGAGGACAG	GATGCTGGAACACAGAATG	63.9	GCTGGGTTCCTCCAGTAT-FAM	63.9	101	1
	rs587739978	-/G						
	rs9279904	AT/-						
	rs139765606	A/-						
	rs146682150	-/A						
52	rs531139227	AAA/-	GGAAAGATACGATGGTAAAAG	62.1	AGTTTTTGGATTTCTGTCAT-HEX	60.2	239	4
	rs140779686	-/A						
	rs148817405	-/T						
	rs67106675	A/-						
